# Impact of PD-1 gene polymorphism and its interaction with tea drinking on susceptibility to tuberculosis

**DOI:** 10.1017/S0950268821000042

**Published:** 2021-01-13

**Authors:** Jing Wang, Mian Wang, Zihao Li, Xinyin Wu, Xian Zhang, Abesig Julius, Hua Zhong, Xin Huang, Mengshi Chen, Hongzhuan Tan, Hengzhong Yi

**Affiliations:** 1 Department of Epidemiology and Health Statistics, Xiangya School of Public Health, Central South University, Changsha, China; 2 Department of Occupational and Environmental Hygiene, Xiangya School of Public Health, Central South University, Changsha, China; 3 Department of Cardiology, Xiangya Hospital, Central South University, Changsha, China; 4 Department of Epidemiology and Health Statistics, Hunan Normal University, Changsha, China; 5 Hunan Provincial Key Laboratory of Clinical Epidemiology, Central South University, Changsha, China; 6 Hunan Institute of Tuberculosis Prevention and Treatment, Changsha, China

**Keywords:** Gene–environment interaction, PD-1, RERI, tea drinking, tuberculosis

## Abstract

The aim of this study was to explore the impact of polymorphism of PD-1 gene and its interaction with tea drinking on susceptibility to tuberculosis (TB). A total of 503 patients with TB and 494 controls were enrolled in this case–control study. Three single-nucleotide polymorphisms of PD-1 (rs7568402, rs2227982 and rs36084323) were genotyped and unconditional logistic regression analysis was used to identify the association between PD-1 polymorphism and TB, while marginal structural linear odds models were used to estimate the interactions. Genotypes GA (OR 1.434), AA (OR 1.891) and GA + AA (OR 1.493) at rs7568402 were more prevalent in the TB patients than in the controls (*P* < 0.05). The relative excess risk of interaction (RERI) between rs7568402 of PD-1 genes and tea drinking was −0.3856 (95% confidence interval −0.7920 to −0.0209, *P* < 0.05), which showed a negative interaction. However, the RERIs between tea drinking and both rs2227982 and rs36084323 of PD-1 genes were not statistically significant. Our data demonstrate that rs7568402 of PD-1 genes was associated with susceptibility to TB, and there was a significant negative interaction between rs7568402 and tea drinking. Therefore, preventive measures through promoting the consumption of tea should be emphasised in the high-risk populations.

## Introduction

Tuberculosis (TB) is one of the top 10 causes of death, with an estimated 10.0 million new (incident) cases and 1.451 million deaths from TB worldwide in 2018. Despite substantial reductions in the prevalence and case fatality rate of TB (more than 50%) since 2000 in the country, China has the second highest TB burden in the world. According to the Global Tuberculosis Report 2019 by the World Health Organization [1], TB incidence in China in 2018 was 61/100 000, accounting for 9% of all estimated incident cases worldwide. Approximately 1.7 billion people, 23% of the world's population, are estimated to have a latent TB infection, while 5–10% of these infected people will develop TB during their lifetime [2], indicating remarkable individual differences, which may be related to the complex interplay of heredity, biology and environment.

Tea drinking is a well-established social determinant known to influence the TB risk. Several studies, including our previous research, have confirmed that there is a significant negative association between tea drinking and TB [3–5], and the mechanisms of the factor on TB have been described elsewhere. Briefly, tea polyphenols, especially epigallocatechin-3-gallate, have been demonstrated to protect the immune system and prevent various pathological processes, including TB infection due to its abilities of antioxidant and free radical scavenging [3–5].

Several studies have shown that the clinical progress and prognosis of TB largely depend on the balance between protective immunity and immunopathological damage of T cells, which is a highly regulated process requiring stimulation from not only the T-cell receptor-major histocompatibility complex but also costimulatory signals (negative or positive) [6]. Programmed cell death-1 (PD-1) is a well-defined co-inhibitory regulator of T cells and can be expressed by a variety of immune cells including T lymphocytes, B lymphocytes, natural killer cells, activated monocytes, dendritic cells and macrophages [7, 8]. In a healthy individual, engagement of PD-1 by its ligands PD-L1 or PD-L2 accelerates the apoptosis of activated lymphocytes, thereby helping in the control of immune tolerance and avoiding the emergence of auto-immunity caused by excessive activation of the immune system [9, 10]. In chronic viral infections, PD-1 is selectively upregulated on the anti-viral effector T cells during the persistent exposure to antigens, inducing T cells to lose functionality and proliferation capabilities [11].

Single-nucleotide polymorphism (SNP), the most common type of polymorphism involving variation at a single base pair, plays a vital role in the transcription and translation of genes and has an association with the occurrence and development of many diseases [12, 13]. More than 30 SNPs have currently been identified in the human PD-1 gene [14], and several of them have been proven to be associated with the susceptibility of numerous diseases, including systemic lupus erythematosus (SLE), rheumatoid arthritis, ankylosing spondylitis, allergic bronchial asthma, type 1 diabetes, multiple sclerosis, acute anterior uveitis, chronic hepatitis B virus infection, subacute sclerosing panencephalitis, antisperm antibody-related infertility and cancers [12, 13, 15–23]. Although the relevance of genetic polymorphisms of PD-1 to TB infection has been investigated previously, the findings showed poor consistency between studies. Guo *et al*. [24] performed an analysis of the association between PD-1 gene and the risk of TB in the Chinese Han population and observed significant associations between SNPs of rs2227981 and rs2227982 sites in PD-1 gene and pulmonary TB susceptibility and clinical features, while some conflicting results were achieved by Li *et al*. [25], who reported that SNPs of rs2227981, rs11568821 and rs10204525 were not associated with susceptibility to TB in Xinjiang Uygur TB patients. Furthermore, Wang *et al*. [26] reported that five SNPs, including rs41435650, rs28539662, rs13023138, rs75565781 and rs36084323 sites in PD-1 were not associated with susceptibility to TB in the Han population. Because the literature discussion is limited and no definitive conclusions have been reached on the association between PD-1 polymorphisms and TB, further investigations are needed to deepen the understanding of the role of these SNPs in the susceptibility to TB in the Han population. Moreover, the SNP sites they analysed were insufficient, and other sites were waiting to be examined. Our previous research found that tea drinking was a protective factor against TB, and increasing tea consumption was associated with a decreased risk of TB [5]. However, it remains unclear whether there is any interaction on TB risk between environmental factors and PD-1 in China.

Hence, this case–control study was designed to detect the polymorphism of the rs2227982, rs36084323 and rs7568402 of PD-1 genes, as well as their interactions with tea drinking, respectively, to determine their joint effects on the susceptibility to TB.

## Methods

### Sources of cases

Two-stage sampling method was used to randomly select four county-level Center for Disease Control and Prevention (CDCs) (i.e. Qidong County CDC, Yueyanglou District CDC, Yueyang County CDC and Hongjiang City CDC) using the random number table among 122 counties/cities/districts in Hunan Province, and then randomly select cases from all TB patients newly registered by the four CDCs. All the TB patients were diagnosed following the TB diagnosis criteria developed by the Chinese Ministry of Health. For microbiologically confirmed TB cases, at least one of the following criterion had to be met: (1) isolation of acid-fast bacilli (AFB) from sputum, gastric aspirate or broncho-alveolar lavage, etc.; (2) sputum culture-positive for Mycobacterium tuberculosis (MTB); or (3) positive results for Xpert MTB/RIF assay. For patients without microbiological evidence, disease diagnosis based on clinical, radiologic manifestations to exclude other diseases, together with the patient's strong positive purified protein derivative (PPD) skin test and confirmed responsiveness to anti-TB treatment after 1 month of follow-up.

### Sources of controls

The two-stage sampling method was also used to randomly select one community health service centre (i.e. Xingang Community Health Service Centre) using the random number table among 14 community health service centres in Kaifu District, Changsha City, and then randomly select one community (i.e. Xin'ansi Community) from six communities covered by Xingang Community Health Service Centre. Because the ratio of male-to-female TB patients was approximately 2.5:1 in Hunan, the controls were selected from permanent residents in the community during the same time and frequency-matched with cases by age and sex. All the controls were confirmed with a history of contact with MTB. For controls with Bacillus Calmette-Guérin (BCG) scar, the average diameter of PPD induration was ⩾10 mm, while for those without BCG scar and no history of BCG vaccination, the average diameter of PPD induration was ⩾5 mm. No abnormalities were found in their chest X-rays.

Both cases and controls were selected from the Chinese Han population without kinship. Individuals aged <18 years, who were known to have low immunity, such as those with HIV infection, severe malnutrition, primary immunodeficiency, long-term use of corticosteroid or immunosuppressant therapy, cancer patients, diabetics, and transplant recipients, were excluded from the study.

### Sample size estimation

Based on an estimated minor allele frequency (MAF) of 0.26 in rs7568402 locus, we estimated that the sample size was 449 subjects for the case and control group, respectively (OR = 1.6, *α* = 0.05, and two-sided, *β* = 0.10, unpaired case–control design).

### Selection of SNPs and genotyping

Three PD-1 SNP sites were selected from the National Centre for Biotechnology Information (NCBI) database. The selection of SNPs was mainly based on the following criteria: (1) SNPs that are associated with susceptibility to TB according to existing literature; (2) SNPs that are associated with the occurrence or development of other infectious diseases according to existing literature. The SNPs with a MAF ⩾5% in the Chinese Han population were included.

Mass spectrometry technology was used to genotype the rs7568402, rs2227982 and rs36084323 of PD-1 genes. The site sequence of rs7568402, rs2227982 and rs36084323 of PD-1 genes were identified in the gene bank, and appropriate primers were designed using Assay Design 3.1 (Sequenom), the quality of which was inspected using matrix-assisted laser desorption ionisation time-of-flight mass spectrometry (MALDI-TOF). The PCR reaction system was 5 μl, including 1.8 μl of ddH_2_O, 0.5 μl of 10×PCR buffer, 0.4 μl of MgCl_2_ (25 mM), 0.1 μl of dNTP (25 mM), 0.2 μl of Hotstar, 1 μl of PCR primer mix and 1 μl of gDNA (20–50 ng). The reaction condition was 95 °C pre-degeneration for 2 min, amplification (95 °C for 30 s, 56 °C for 30 s, 72 °C for 60 s) for 45 cycles, and 72 °C extension for 5 min. The enzyme digestion reaction system was 2 μl, including 1.53 μl of ddH_2_O, 0.17 μl of SAP buffer and 0.3 μl of SAP enzyme; the reaction condition was 37 °C for 40 min and 85 °C for 5 min. The single base extension reaction system was 2 μl, including 0.619 μl of ddH_2_O, 0.2 μl of iPLEX buffer, 0.2 μl of terminator mix, 0.94 μl of extend primer mix and 0.041 μl of iPLEX enzyme; the corresponding reaction condition was 94 °C pre-degeneration for 30 s, 40 cycles of amplification (5 cycles of three temperature settings: 94 °C for 5 s, 52 °C for 5 s, 80 °C for 5 s and 72 °C extension for 3 min). Then, the resin was purified by plating the clean resin into a 6 mg resin plate, and the resin-extended extension product was transferred to a 384-well SpectroCHIP (Sequenom) chip for spotting (MassARRAY Nanodispenser RS1000). The difference in bases caused by SNP polymorphism could be converted into molecular weight difference using Sequenom MassARRAY® SNP assay. The molecular weight of the extension product was detected by MALDI-TOF, and the analysis was performed using MassArray TYPER 4.0. The difference in molecular weight could be used to determine the SNP typing.

### Information and sample collection

Self-designed questionnaire was used to collect data for demographic characteristics (sex, age, marital status, educational background, etc.) and environmental factors (history of BCG vaccination, tea drinking, etc.). Exposure to tea drinking was defined as at least one cup of tea drinking per week on average for over 6 months. In the survey, the respondents were asked if they are regular tea drinkers, including green tea, black tea or home-made smoked tea. If so, the major concerns then centred around their tea drinking habits, including the amount of tea consumed per consumption period, frequency, duration and tea leaf choices. Weight was measured to 0.1 kg using a digital bathroom scale, and height was measured in centimetres to 0.1 cm using a height meter. Body mass index (BMI) was calculated and the results were divided into three groups (⩽18.5, 18.5−24.9, ⩾25 kg/m^2^). In addition, participants were examined carefully by trained doctors to measure the diameter of BCG scar, and those with a diameter of no less than 3 mm were judged to have a history of BCG vaccination.

Five-millilitre peripheral blood specimens were aseptically collected in EDTA anticoagulant tubes from each participant after overnight fasting and stored at 4 °C before use. Genomic DNA was extracted from peripheral blood leucocytes using a Wizard Genomic DNA purification Kit (Promega, Shanghai Sangon Biotech Co., Ltd), and the quality-controlled DNA was stored at −80 °C until assay.

### Statistical analysis

EpiData 3.0 was used to input data, and SPSS 22.0, SAS 9.2 and R 3.5.3 software were used for data analysis. Categorical data were expressed as frequency and percentage. The *χ*^2^ test was conducted to compare grouped data and detect Hardy–Weinberg equilibrium. Logistic regression was used for multivariate analysis. To exclude possible confounding risk factors, the occurrence of TB was used as the dependent variable, rs7568402, rs2227982 and rs36084323 of PD-1 genes were used as the independent variables, and the sex, age, marital status, educational background, BMI, BCG vaccination history, smoking status, alcohol drinking and tea drinking were used as the covariates, and multivariate unconditional logistic regression analysis was conducted. The interaction of additive effects between the SNP and tea drinking was analysed, and the relative excess risk of interaction (RERI) was estimated if the main effect on TB was meaningful. Marginal structural linear odds models [27] were used for point estimation and interval estimation of RERI. RERI>0 indicates positive interactions. All tests of hypotheses were two-tailed with a type 1 error rate fixed at 5%.

## Results

The study participants included 503 TB patients and 494 controls. The distributions of demographic characteristics and associated risk factors between the TB patient group and the control group have been reported in our previous study [28]. The two groups exhibited no statistical difference (*P* > 0.05) in terms of sex, age, marital status, educational background and alcohol drinking, while differences in BMI, history of BCG vaccination, smoking status and tea drinking were statistically significant (*P* < 0.05).

Hardy–Weinberg equilibrium test showed genetic equilibrium (*P* > 0.05) for rs2227982, rs36084323 and rs7568402 of PD-1 genes in the control group (*χ*^2^ = 3.690, 2.399, 0.642, respectively, *P* > 0.05), which indicated that the three loci of PD-1 genes were evenly distributed in the control group, and the samples were all from the same Mendelian population (Table 1).

**Table 1.** Genotypes of the PD-1 genes in two groups

		TB patients		Controls		*χ*^2^	*P*-value
		*n*	%	*n*	%		
rs2227982	GG	128	25.45	131	26.52	0.171	0.918
	GA	274	54.47	267	54.05		
	AA	101	20.08	96	19.43		
HWE-P				0.055			
rs36084323	AA	143	28.43	122	24.70	1.921	0.383
	AG	260	51.69	264	53.44		
	GG	100	19.88	108	21.86		
HWE-P				0.121			
rs7568402	GG	223	44.33	270	54.66	12.037	0.002*
	GA	233	46.32	195	39.47		
	AA	47	9.34	29	5.87		
HWE-P				0.423			

HWE-P, Hardy–Weinberg equilibrium-*P* value.

*P < 0.05.

The univariate analysis showed that the genotypes of rs7568402 locus located in PD-1 gene were associated with susceptibility to TB (*χ*^2^ = 12.037, *P* < 0.05), while no statistically significant differences were observed in the genotypes of rs2227982 and rs36084323 locus between the TB patients group and the controls group (*P* > 0.05). After adjusting for the covariates of sex, age, marital status, educational background, BMI, smoking status, alcohol drinking, tea drinking and history of BCG vaccination, multivariate unconditional logistic regression analysis showed that rs7568402 of PD-1 genes was associated with an increased risk of TB. Genotypes GA, AA and GA + AA at rs7568402 (compared with genotype GG) were more prevalent in the TB patient group than those in the control group (*P* < 0.05), with OR being 1.434, 1.891 and 1.493, respectively (Table 2).

**Table 2.** PD-1 gene polymorphism *vs.* TB incidence

		TB patients		Controls		OR_c_（95% CI）	OR_a_^#^（95% CI）
		*n*	%	*n*	%		
rs2227982	GG	128	25.45	131	26.52	1 (reference)	1 (reference)
	GA	274	54.47	267	54.05	1.050 (0.781–1.412)	1.036 (0.762–1.407)
	AA	101	20.08	96	19.43	1.077 (0.743–1.560)	1.065 (0.724–1.567)
	GA + AA	375	74.55	363	73.48	1.057 (0.797–1.403)	1.043 (0.778–1.399)
rs36084323	AA	143	28.43	122	24.70	1 (reference)	1 (reference)
	AG	260	51.69	264	53.44	0.840 (0.625–1.130)	0.820 (0.603–1.113)
	GG	100	19.88	108	21.86	0.790 (0.549–1.137)	0.768 (0.526–1.120)
	AG + GG	360	71.57	372	75.3	0.826 (0.623–1.094)	0.805 (0.601–1.077)
rs7568402	GG	223	44.33	270	54.66	1 (reference)	1 (reference)
	GA	233	46.32	195	39.47	1.447 (1.115–1.876)	1.434 (1.100–1.869)*
	AA	47	9.34	29	5.87	1.962 (1.195–3.221)	1.891 (1.141–3.135)*
	GA + AA	280	55.66	224	45.34	1.513 (1.179–1.943)	1.493 (1.158–1.926)*

^#^Multivariate logistic regression model was used to adjust the covariates of sex, age, marital status, educational background, BMI, smoking status, alcohol drinking, tea drinking and BCG vaccination.

*P < 0.05.

Marginal structural linear odds models were used to analyse the impact of the interactions between PD-1 genes and tea drinking on susceptibility to TB. Adjusting for the covariates of sex, age, marital status, educational background, BMI, alcohol drinking, smoking status and history of BCG vaccination, the RERI between rs7568402 of PD-1 genes and tea drinking was found to be −0.3856 (95% confidence interval (CI) −0.7920 to −0.0209, *P* < 0.05) (Table 3), which suggests negative interactions. However, the RERI between rs2227982 of PD-1 genes and tea drinking was not statistically significant (0.1228; 95% CI −0.1118 to 0.3574). Similar findings were observed between rs36084323 of PD-1 genes and tea drinking, with the adjusted RERI of 0.1851 (95% CI −0.0571 to 0.4271).

**Table 3.** Impact of interaction between rs7568402 and tea drinking on the incidence of TB

Tea drinking	Group	GG		GA + AA		OR	95% CI
		*n*	%	*n*	%		
No	TB patients	116	45.31	140	54.69	1.402	0.967–2.031
	Controls	108	53.73	93	46.27		
Yes	TB patients	107	43.32	140	56.68	1.618	1.150–2.276
	Controls	162	55.29	131	44.71		
RERI_c_	−0.0215 (−0.5564, 0.5134)						
RERI_a_^#^	−0.3856 (−0.7920, −0.0209)*						

^#^Adjusted for the covariates of sex, age, marital status, educational background, BMI, smoking status, alcohol drinking and BCG vaccination.

*P < 0.05, RERI < 0 suggests negative interactions.

## Discussion

TB is a relatively common infectious disease with high morbidity and high susceptibility of being chronic in China. The aetiology of TB is very complicated, involving both environmental and genetic factors. In addition, it has been proposed that there may be multifactorial interactions between a number of risk factors and genetic abnormalities. The present study was made to investigate the polymorphisms of PD-1 genes as well as their combined effects with environmental factors on the susceptibility to TB.

The findings indicated that mutation of rs7568402 was a predisposing factor of TB infection, while mutations of rs2227982 and rs36084323 did not exhibit such an association, which was consistent with a prior study conducted by Wang *et al*. [26], comprising 100 TB patients and 100 healthy volunteers from the Chinese Han population. However, another study involving 262 TB patients and 255 healthy volunteers observed significant associations between rs2227982 and TB susceptibility and its clinical features [24]. The discrepancy in the results may be attributed to differences in the study design, including the sample size, study population, selection of SNPs and characteristic reasons of different regions. Moreover, the possible impacts of non-genetic factors were not adjusted in these studies, which might weaken the validity of the evidence. Considering the effective controlling for potential confounders, such as sex, age, marital status, educational background, BMI, smoking, alcohol drinking, tea drinking and history of BCG vaccination in the present study, our study results are more convincing.

Furthermore, according to the results of the marginal structural linear odds models, we discovered a negative interaction between rs7568402 of PD-1 genes and tea drinking, which indicated that tea drinking, consistent with our prior studies [5, 28], was a protective factor in TB infection. Moreover, black tea, oolong and green tea were all confirmed to be inversely associated with TB and significant dose–response relationship was discovered [5]. Experimental studies also showed that increasing tea consumption could raise blood catechin levels, leading to increased plasma antioxidant activity and resistance to diseases [29]. Furthermore, tea polyphenols have been proposed to be responsible for the inverse relation due to their abilities of antioxidant and free radical scavenging anti-inflammation effect [4, 28]. The polyphenol content of tea differs from different degree of fermentation and an inverse association is exhibited between the two variants. Green tea is non-fermented and oolong tea partially fermented, while black tea and smoked tea are fully fermented. Even so, theaflavins in black tea is confirmed to protect against oxidative stress, which supports observations in our present study [30]. On the contrary, studies showed that compared with healthy subjects, patients with active pulmonary TB had significantly higher inflammatory cytokine levels [31]. Mutations of rs7568402 (GA, AA and GA + AA), which is located in the 3′ untranslated region, may alter the inflammatory cytokine levels through modulating polyadenylation [32]. In addition, since studies have reported that untranslated region alternative polyadenylation (UTR-APA) may lead to the alteration of stability, localisation or protein translation efficiency in the mRNAs [33, 34], we hypothesised that mutations of rs7568402 may affect the transcription and activation of the PD-1 genes through UTR-APA, which perhaps further affect the immune response through the PD-1/PD-L1 signalling pathway and consequently influence the progression of TB infection. Nevertheless, the exact biological mechanism remains to be determined.

Overall, considering that the mutation in rs7568402 was proved to be a predisposing factor for TB in univariate analysis, we hypothesised that the antagonism of tea drinking on TB disease was much greater than the potentiation of mutations in rs7568402 and thus helped in decreasing the risk for TB as a whole. This might indicate that the behavioural intervention of encouraging drinking tea may not only lower TB incidence for patients with mutations in rs7568402 from the perspective of the main effect, but also improve the prognosis by utilizing such a negative interaction.

In the present study, no interactions were observed between both rs36084323 and rs2227982 of PD-1 and tea drinking on TB risk. In the case of rs36084323, it is located in the promoter region of PD-1 and mutations of rs36084323 might influence the transcription and activation of the PD-1 gene [13], while rs2227982 is located in the 5th exon, whose mutations would lead to a non-synonymous mutation and cause an amino acid substitution during protein synthesis, which may result in the functional variations of PD-1 [13, 35]. Numerous studies have investigated the association between these polymorphisms and the risk of various human diseases; however, the findings remain discrepant. The rs36084323 polymorphisms have been assumed as a risk factor for NSCLC in a Japanese population [36] and associated with breast cancer in a Chinese group [37]. Moreover, it has been reported that rs36084323 and rs10204525 polymorphisms combined with chronic HBV infection contribute to the development of HCC in a Chinese population [38]. While a meta-analysis conducted by Da *et al*. [39] revealed no significant association between PD-1 rs36084323 polymorphism and overall cancer susceptibility. Rs2227982 was demonstrated to be associated with the risk and disease progression of SLE, allergic bronchial asthma, AS, breast cancer, and gastric and digestive system cancers, such as adenocarcinoma, in previous reports [13, 21]. A recent study indicated a significant association between rs2227982 and HBV infection as well as a gene–gene interaction with rs10204525 in a Chinese population [21]. On the contrary, a meta-analysis involving 12 case–control studies revealed no associations between cancer risks and rs2227982 in all genetic models and alleles [35].

To the best of our knowledge, rs7568402 has previously not been associated with any known autoimmune disease and cancer. In a case–control study conducted by Tejeda *et al*. [32], a significant association between systemic JIA and rs7568402 was observed after stratification by JIA categories but was not replicated in an independent multi-ethnic systemic JIA cohort. Therefore, the study concluded that there was no association between PD-1 rs7568402 and systemic JIA.

It should be noted that this study has some limitations. First, we detected limited polymorphisms in PD-1; therefore, it remains to be determined for the analysis of gene–gene interactions and gene–environment interactions among other polymorphisms of PD-1 on TB susceptibility. Second, all the participants have been limited to Han Chinese, and genetic deviations may exist among different ethnic populations. In future studies, we will expand the sample size and verify the results of this study with data from other populations. Third, we did not distinguish the type of tea and consider tea drinking habits such as the concentration of intake and the resulting blood levels of catechin in the analysis. These could make the accuracy of our estimation on the interactions compromised, and further investigations in these aspects are needed to elucidate the effect of tea drinking and its interactions with PD-1 polymorphisms on TB more comprehensively. Fourth, because the study design was a case–control study, the demonstration of causality in our study is limited. Herein, the results need to be further verified in cohort studies in the future. However, all the cases selected in this study were new cases, and the main factors (PD-1 polymorphisms and tea drinking) of the study were relatively stable, so we believe recall bias had little effect on the results. Additionally, because all the possible impacts of non-genetic factors were adjusted, the results observed in our study should be reliable.

The study mainly explored the associations from an epidemiological perspective, and investigations on the biological mechanism of variants with positive outcomes in the PD-1 gene will be important directions for future research. Additionally, the interactions between different genes or different loci on the same gene should be considered to reveal the true association between genes and diseases as well as the pathogenesis of TB from a new perspective.

## Conclusions

Rs7568402 polymorphism of PD-1 affected the host's susceptibility to TB infection, and there was a significant negative interaction between rs7568402 of PD-1 genes and tea drinking on susceptibility to TB. This finding is significant for identifying populations with a high risk of developing TB, and suggests that tea, as a highly affordable and widely accepted beverage, may reduce the risk of TB in the high-risk groups. Therefore, preventive measures through promoting the consumption of tea as the daily drink should be emphasised in the high-risk populations.

## Data Availability

The data that support the findings of this study are available from the corresponding author, Chen M, upon reasonable request.
